# Purpura, Occurring in Nervous Diseases

**Published:** 1878-06

**Authors:** J. B. Andrews

**Affiliations:** First-Assistant Physician N. Y. State Lunatic Asylum, Utica, N. Y.


BUFFALO

Medical and Surgical Journal.

VOL. XVII. JUNE, 1878. No. 11.

### Original Communications.

—:o:—

ART. I.—*Purpura—Occurring in Nervous Diseases.* By J. B. ANDREWS, A. M., M. D., First-Assistant Physician N. Y. State Lunatic Asylum, Utica, N. Y.

Purpura has been defined as a painless, passive exudation of blood into the substance of the skin. There are three forms usually recognized, viz.: the simple, hæmorrhagic and scorbutic, distinguished from each other by well defined characteristics. In the simple form there are petechiæ or ecchymoses more or less extensive in the dermal substance. In the hæmorrhagic form there is bleeding from the mucous surfaces. Scorbutus is a disease dependent upon known causes, with definite symptoms and well established methods of treatment.

Purpura, as it is generally met with, has been attributed to a blood dyscrasia, in which there is a change in the constituents which renders the blood more fluid than in the normal, healthy state, and destroys its coagulability. This explanation, however, will not suffice in the cases which are reported in this article, as the conditions productive of blood cachexia did not exist. They are considered as instances of trophic lesions from vaso-motor disturbance. That this lesion is sufficient to account, not only for serous and hæmorrhagic effusions or exudations, but also for destruction of tissues is susceptible of illustration bordering upon absolute proof. Some of these with authorities will be mentioned.

There are forms of hæmorrhagic exudation or effusion which are not classified under the name Purpura. The rupture of vessels by the application of external violence produces a bruise or ecchymosis. Of the same character are the petechiæ seen upon the forehead, face, neck or chest of epileptics, immediately after a fit, the result of rupture of capillaries from blood pressure during a convulsion, and which are sometimes so marked as to be an aid in diagnosis of the disease.

Another example, due to an entirely different cause, occurs frequently in the practice of the physician, with a history somewhat as follows: The patient awakes in the morning, and to his surprise discovers an ecchymosis in the conjunctiva, usually of one eye. This may be slight, or so extensive as to give the cornea the appearance of an excavation in the midst of a recent hæmorrhage. Occasionally this follows a period of severe exertion or exposure to cold, but there is often no cause apparent in the circumstances or surroundings of the individual. The explanation is found in the local paralysis of the vaso-motor nerve that controls the circulation of blood, thus allowing a dilatation of the vessel until rupture occurs. Other examples to be attributed to nervous defect, are found in persons in ordinary health, as in females during menstruation when purpuric spots appear upon the neck, arms or back.

There are cases of purpura so closely connected with certain diseases as to derive their names from them as Purpura Rheumatica, Neurotic Purpura. The latter is associated with neuralgia, and instances of it have been reported by Prof. S. Weir Mitchell, of Philadelphia, (*Am. Journal Med. Sciences,* April, 1869,) and by Dr. Tyrrall, of California, (*Pacific Med. and Surg. Journal,* June, 1876). The purpuric spots were thought to be the final evidence of nerve irritation and paresis, which, affecting the nutrition of the blood vessels, through the vaso-motor nerves, allowed extravasation of blood to take place under the cuticle, forming the patches of ecchymosis, which were such a prominent characteristic in the cases mentioned. In their origin and progress they would seem to sustain the name given them of "Neurotic Purpura," and to increase the number of skin affections which medical science has classified under the neuroses.

As showing the influence of disturbed conditions of the sympathetic system, reference may be made to the congestion of the face and neck after section of the cervical sympathetic. The same condition is observed in the face, lips and extremities in cases of advanced insanity, especially in dementia, and this may even lead to rupture of the vessels.

Cases of Purpura occurring in the insane were reported by Dr. J. C. Bucknill, in the *Journal of Mental Science*, in 1855. Other observers have given instances in which the spots covered large portions of the body and resembled severe and extensive bruises. They occurred in patients with chronic insanity and paralysis, debilitated and cachectic, but not scorbutic. A transcript is presented of a few cases of like character.

Case I.—Woman, 29 years of age, who had been an inmate of the State Asylum at Utica for more than five years. When admitted she was a marked case of melancholia, but soon became demented and continued dull, indifferent to her surroundings, and manifested little mental or physical activity. She retained flesh and strength and took food regularly at the table, though inclined to restrict her diet to food of the amylaceous order. This was supplemented by milk, and though she received a fair amount of food, she was during the whole period anæmic. In September, 1875, without any mental or physical change purpura was suddenly developed. It was located upon the outer side of the left thigh, and extended from the hips downwards to the ankles, and on the under aspect of the thigh from the line of pressure made by the edge of a chair when sitting. Below the knee, and especially about the ankle, the condition was equally marked and accompanied by an extensive serous exudation. There was no complaint of pain or of discomfort, and no constitutional disturbance. The gums were firm and hard, though pale. The patient was put to bed and given liquid foods, stimulants and mineral acids. The ecchymosed spots passed through the changes in color common to hæmorrhagic effusions under the skin, and entirely disappeared after about six weeks. There was no return of the condition, though the debility of the patient increased, and she died of phthisis in February, 1876.

Case II.—Woman, 35 years of age, who had been insane three years, and an inmate of the Asylum fifteen months. She was excitable, irritable, talkative, and had periods of maniacal noise and violence, in which she resisted care and refused to take food, or medicines prescribed, and was also anæmic. One morning she complained of the spots on her limbs, and of a soreness of her flesh as if she had been bruised, though she asserted she had not been injured in any way. There was no constitutional disturbance. The effusion was nearly as extensive as in the other case, but was distributed entirely upon the outer aspect of the thighs. There was a marked change in the mental condition of the patient. She became quiet, docile, and yielded readily to every suggestion regarding her care and treatment. She was treated in the same way, with liquid food, stimulants and mineral acids, and in about one month recovered and returned to her former irritable and excitable condition.

Case III.—Man, age 33 ; had been insane for more than three years, and an inmate of the Asylum but a few months. He was considerably reduced, but, after admission, ate well, and gained in flesh. In the spring of 1876, under the influence of delusion he began to refuse food, except bread and water ; food was, however, administered to him in sufficient variety and quantity, but after some two weeks Purpura was developed, a patch as large as the hand appearing upon the inner side of the thigh. Food was continued by the stomach tube and mineral acid was added. Recovery from the condition rapidly followed, and though the patient has since refused food and been equally debilitated, there has been no recurrence of the condition.

Case IV.—Man, age 37 ; had been insane for about one year ; had melancholia with syphilis, and had been in the Asylum but a short time. During some of the earlier months of his attack he ate voluntarily only bread and water, but after his admission he took a greater variety of food and gained materially in flesh and general condition. At this time he again refused food, which was administered by stomach tube. After some two weeks, purpuric spots appeared upon the inner side of the thigh and leg; they were some four inches long, and nearly the same in width. To the liquid diet and mineral acid the biniodide of mercury was added. In three weeks the ecchymosed spots had entirely disappeared, and although for some time the patient ate sparingly and demanded watchfulness to insure the ingestion of the proper amount of food there was no further developement of the condition.

In the latter case a microscopic examination of the blood was made by Mr. Deecke, the Special Pathologist of the Asylum. It was thought that this might throw some light upon the etiology of the diseased state. There was, however, no disturbance of the relations of the white to the red corpuscles in the blood, and the only change noticed was that the corpuscles had lost their distinct numular shape, and had acquired a more marked spherical form.

The two following are given as instances of Purpura Hæmorrhagica:

Case V.—*Purpura Hæmorrhagica.*—Woman, age about 45; had lived in comfortable circumstances, and was in good flesh and fully nourished. She became insane some four months before admission; was a mild case of melancholia, induced by ill-health from grief and anxiety. Soon after coming to the Asylum she refused food and ate so sparingly that it was necessary to urge her to take a proper amount. About a month after admission she went to bed complaining of being sick, and for two days her temperature was somewhat elevated. This declined, and there followed in some four days an exudation of blood from the gums, fauces and nasal membrane. Petechiæ appeared about the face and chest, and these were succeeded by spots of Purpura, of irregular size up to several inches in area, which covered much of the dermal surface of the body and extremities. On the fifth day there was an extensive hæmorrhage from the lungs, of which prior examination had revealed no diseased condition. This reduced her to a point where her life was despaired of. At this time she began to take food freely, and was given stimulants and mineral acids.

Under these she has rallied, and is now up and able to be about, though there remains a consolidation of the lung substance, which is no doubt due to the hæmorrhage. Another point of special interest is the formation of small abscesses, which mark the petechial spots.

Case VI.—Man, age 26; had been wild and dissolute and was of quarrelsome, irritable disposition. In a debauch some two years before admission to the Asylum he received a blow over the right temporal region which produced unconsciousness for a short time. About six months after this there were evidences of mental disturbance. He was feeble in mind, had exalted delusions of wealth, and at times marked disturbance of speech. He continued quiet and harmless for some eighteen months, when he became noisy, violent and maniacal, and on this account was sent to the institution He lived about six months, and during the whole period was restless, noisy, incoherent, had hallucinations of sight and hearing, hesitancy of speech, and extravagant delusions of wealth and power. He had no convulsions, ate heartily and body was well nourished. Two weeks before his death the urine passed was highly colored and apparently sanguinolent. On examination it was found to be strongly alkaline, and to contain blood and pus in large quantitiy. The urine continued thus for four or five days, when it regained the normal condition. There was no constitutional disturbance and no evidence of disease of the bladder or kidneys. He died suddenly from hæmorrhage into upper part of cord. *Autopsy.* Rupture of the vertebral artery, which contained a thrombus and showed an aneurismal dilatation one-half inch below the basilar artery ; slight pachy-meningitis interna over the right temporal, the right parietal and a part of the right central convolutions. The right side of the cerebrum showed the following softened spots: one of the size of a silver five cent piece in the first temporal convolution near the ascending branch of the Sylvian fissure; one of the same size in the second temporal convolution above the second temporal fissure; one of irregular shape, about one-eighth to one-fourth of an inch, by seven-eighths of an inch in the second temporal convolution, above the middle temporal fissure; one of about the same shape but still a little larger than the foregoing in the third temporal convolution, above the anterior part of the lower temporal fissure The thoracic and abdominal, including the urinary, organs, to which attention was especially directed, were in a healthy state.

This case, though in many features resembling Paresis, was one of brain softening. It is reported as illustrating a class of cases in which the hæmorrhagic effusion is symptomatic of irritation from cerebral lesion.

There are certain points of resemblance in the cases here reported. In none of them did the conditions under which purpura is ordinarily developed exist. The hygienic surroundings were of the most favorable character, and none of them suffered from exposure to damp or cold. Food in proper quantity and quality, except at short intervals, was taken, and when refused was administered. They were, with one exception, cases of chronic insanity, and all were suffering from disease of the central nervous system—a condition in which there is often disturbance of the sympathetic as well.

The influence of disorder of the vaso-motor nerves in producing purpura in certain conditions and nervous diseases, as in neuralgia has already been stated. That more extensive lesions may depend upon this cause is shown by experiment. Axman, as reported by Simon, has caused extravasation of blood by interrupting the nervous supply in parts. He destroyed several ganglia of the sympathetic nerve of a frog, and the result was not only hæmorrhage but softening of the tissues of the parts deprived of nervous influence.

Charcot, in his "Lectures on Diseases of the Nervons System," in the chapter "on disorders of nutrition, consecutive on lesions of the spinal cord and brain," gives cases illustrative of trophic lesions, resulting in the destruction of the skin and underlying tissues. These are the acute bed sore, occurring in apoplexy, which is found in the gluteal region and when the paralysis is unilateral always upon the same side, and the acute bed sore of spinal origin, which is found only in the sacral region. The same destruction of tissue frequently accompanies injuries or wounds of the spine, occurring at the same time and consequent upon cerebral lesion. Charcot mentions the passage of sanguinolent urine, alkaline in reaction and sometimes purulent. (See case 6.)

Another example of the effect of nerve lesion is seen most frequently in Paresis or general paralysis of the insane, though sometimes in ordinary paralysis, in the effusion of serum or blood under the dermal surface, which often goes on to the destruction of the deeper tissues, forming extensive and dangerous sloughs. In this form of disease there is not only lesion of the central nervous system, but the whole nervous expansion is involved.

In investigating the cause of nutritive changes, on which the different conditions of congestions, exudations or effusions, and mortification depend, there will be found two theories of special prominence. M. Schiff, in discussing this question, asserts "that the changes of nutrition originate in the hyperæmic parts, in cases of vaso-motor paralysis, under the influence of the slightest local mechanical irritant, and that inflammation here readily takes on a destructive character." Charcot acknowledges that many of the phenomena do directly depend upon either the dilatation or contraction of the smaller vessels determined by nervous influence, and that local nutrition may receive a serious blow from the mere fact that the part has been withdrawn from vaso-motor innervation. He, however, recommends the trophic-nerve theory as better explaining the phenomena observed. This theory presupposes the action of a set of nerves whose office is to control assimilation and absorption, but whose existence even has not been anatomically demonstrated. Some of the views held, with the analogies which sustain them, regarding nutrition changes are thus simply stated. If the cases reported direct attention to the subject, and induce any to record facts occurring within their experience, which may tend to shed additional light upon the question, the purpose of the writer will have been attained.

